# Microinfarcts are Associated with Cognitive Impairment in Neurofibrillary Tangle Predominant Decedents: Evidence from the NACC Autopsy Cohort

**DOI:** 10.21203/rs.3.rs-7036276/v1

**Published:** 2025-07-14

**Authors:** Nicko C. Martinez, Krishna L. Bharani, Saadia Hasan, Cellas A. Hayes

**Affiliations:** University of California, Riverside; Stanford University School of Medicine; Stanford University School of Medicine; Stanford University School of Medicine

**Keywords:** Neurofibrillary tau, microinfarcts, Alzheimer’s disease continuum, vascular neuropathology, cognitive impairment, neuropathology, vascular neuropathology

## Abstract

**Background:**

A growing number of older adults exhibit neurofibrillary tangle pathology without significant amyloid deposition, a biomarker profile consistent with suspected non-Alzheimer’s pathophysiology or primary age-related tauopathy. The cognitive consequences within this subgroup remain poorly characterized, particularly with respect to vascular comorbidity. This study investigates whether vascular neuropathologies are associated with pre-mortem cognitive decline among individuals with predominately neurofibrillary tangles and low to none neuritic plaque pathology detected post-mortem.

**Methods:**

The sample included autopsy-confirmed data from 579 participants in the National Alzheimer’s Coordinating Center (NACC) cohort with intermediate-to-high Braak stage (B2–B3) and absent or minimal neuritic plaques (C0-C1). Vascular neuropathologies included arteriolosclerosis, atherosclerosis of the circle of Willis, gross infarcts/ lacunes, and microinfarcts were assessed for associations with global cognition (Clinical Dementia Rating Sum of Boxes, CDR-SOB) and harmonized cognitive domain specific performance (memory, executive function, and language) using multivariable regression adjusted for age, sex, education, and apolipoprotein ε4 status. Sensitivity analyses further controlled for cardiovascular risk, excluded individuals with any neuritic plaques, and controlling for individual neuritic plaque and Braak staging.

**Results:**

Cross-sectionally, microinfarcts were consistently associated with poorer memory (β = − 0.28, p = 0.02), executive function (β = − 0.24, p = 0.02), and language (β = − 0.21, p = 0.02) approximate to death. Gross infarcts were associated with language impairment and higher CDR scores. These associations remained significant after adjusting for cardiovascular risk and were even stronger when restricted to strictly neuritic amyloid negative individuals.

**Conclusions:**

Microinfarcts may represent a key driver of cognitive impairment in neurofibrillary tangle predominant individuals. These findings highlight a vascular-neurodegenerative pathway that warrants consideration in non-neuritic plaque models of cognitive decline and may inform targeted prevention strategies.

## Introduction

Cognitive decline in late life is increasingly understood as the result of multiple overlapping pathologies (i.e., polypathology) that contribute to mixed dementia, despite the majority of individuals receiving a clinical diagnosis of Alzheimer’s disease (AD) (1–5). Traditionally, AD was clinically defined by the accumulation of amyloid-β (Aβ) plaques (A+) and neurofibrillary tau tangles (T+). However, recent diagnostic frameworks have shifted toward a biological definition of AD, incorporating evidence of amyloid (A), neurofibrillary tau (T), and neurodegeneration (N) to better understand the clinical significance and interactions of distinct AD pathology (6, 7).

Historically, definitive AD diagnosis required autopsy with the identification of the density and spread of Aβ plaques and neurofibrillary tau tangles using immunohistochemical or special stains (8). But with advances in positron emission tomography and cerebrospinal fluid biomarkers, and more recently, plasma-based biomarkers, the clinical diagnosis of AD has become more biologically accurate with technological advancements (6, 7). Despite the central role of amyloid and tau in AD, not all individuals follow a stereotypical pattern of pathology. Some individuals demonstrate intermediate or advanced tau pathology in the absence of amyloid deposition, suggesting alternative mechanisms of neurodegeneration (9, 10). Mounting evidence indicates that vascular neuropathologies accompany AD and Lewy body disease (LBD), including arteriolosclerosis, atherosclerosis, cerebral infarcts, and microvascular injury which can contribute to late-life cognitive decline and dementia both independent of and in consort with AD-related Aβ pathology (11, 12). However, the distinct impact of vascular pathology on cognition, particularly in the context of predominantly neurofibrillary tau and none to low neuritic plaques, remains underexplored.

Large autopsy-based cohort studies have consistently shown that vascular lesions are highly prevalent in aging brains (12, 13). Arteriolosclerosis and atherosclerosis are arguably the most prevalent vascular neuropathologies found at autopsy and also coexist with AD pathology (12, 14). Vascular contributions to cognitive impairment appear to follow distinct mechanistic pathways compared to AD pathology and exerts effects even in individuals with minimal amyloid deposition. Studies have shown that vascular injury may precede or act independently of amyloid, serving as an early and potentially modifiable driver of neurodegeneration (15, 16). For example, recent findings demonstrate that arteriolosclerosis is associated with poorer cognitive outcomes independent of AD neuropathologic change (ADNC) and LBD pathology (12).

Microinfarcts (i.e., small ischemic lesions often undetected on neuroimaging) have been strongly associated with deficits in memory and executive function, despite adjusting for AD pathology (17). Gross infarcts and large-vessel atherosclerosis are also robust predictors of dementia, especially among older adults with low or unclear AD biomarkers (18–20). These findings provide further evidence that vascular pathology may not only interact with traditional neurodegenerative processes but may also independently mimic or drive cognitive impairment in the absence of amyloid pathology.

A growing number of older adults exhibit neurofibrillary tau pathology without amyloid plaques, a biomarker profile often referred to as primary age-related tauopathy (PART) in post-mortem studies or suspected non-Alzheimer’s pathophysiology (SNAP) clinically (9, 10). The cognitive outcomes of this group are not well characterized, and vascular pathology may be a key determinant of symptom expression. Given the high prevalence of vascular disease in aging populations, and its well-established impact on white matter injury, cerebral perfusion, and inflammation, there is a pressing need to better understand vascular contributions to cognitive decline beyond traditional AD pathology accumulation. Doing so may reveal non-amyloid pathways of neurodegeneration and provide opportunities for intervention that have been overlooked in amyloid-centric models of dementia.

In this study, we leveraged harmonized demographic data, autopsied verified neuropathology collection, and harmonized cognitive scores from the National Alzheimer’s Coordinating Center (NACC) autopsy dataset to investigate the role of vascular neuropathologies (arteriolosclerosis, atherosclerosis of the circle of Willis, cerebral amyloid angiopathy, gross infarcts/ lacunes, and microinfarcts) in cognitive outcomes among individuals with intermediate or high neurofibrillary tau pathology but none or low neuritic plaques. We specifically investigated whether vascular neuropathologies significantly contribute to cognitive decline across multiple cognitive domains (memory, executive function, language, and global impairment) among neurofibrillary tangle predominant decedents.

## Methods

### National Alzheimer’s Coordinating Center

In this study, the NACC neuropathology dataset represents a sub-sample from data acquired from the September 2024 (21). The NACC serves as a central repository for standardized research data collected from Alzheimer’s Disease Research Centers (ADRC) across the United States. Funded by the National Institute on Aging, the ADRC began contributing data to NACC in 2005 through regular assessments of enrolled participants. Participants are enrolled across the full spectrum of cognitive function, from cognitively normal to those with mild cognitive impairment (MCI), non-MCI cognitive impairment, and dementia. All participating ADRC obtained approval from their respective institutional review boards prior to data collection. For the present study, the decedents included were from 34 ADRC. The autopsied data set generated from variable (NACCAUTP) yield 8,188 participants.

### Data cleaning and processing

To prepare key neuropathological and genetic variables for analysis, several variables were recoded and cleaned in a harmonized autopsy-confirmed dataset. Apolipoprotein (*APOE*) ε4 carrier status was derived from the NACCAPOE variable, with participants coded as 1 if they carried at least one ε4 allele (e3/e4, e4/e4, or e2/e4) and 0 if they were non-carriers (e2/e2, e2/e3, e3/e3). This method has been similarly used on other NACC neuropathology research (14).

ADRC uploaded data to NACC using neuropathology forms 9 and 10 per consensus guidelines (8, 22). Following previous NACC methodologies and aligning with clinical influence on cognition, the vascular neuropathological markers were categorized as binary: arteriolosclerosis (NACCARTE), atherosclerosis of the circle of Willis (henceforth atherosclerosis) (NACCAVAS), and cerebral amyloid angiopathy (CAA) (NACCAMY) were binarized to indicate presence (moderate or severe pathology) versus absent/ mild (12, 23). Additionally, the variables gross infarcts/ lacunes (henceforth referred to as gross infarcts) (NACCINF) and microinfarcts (NACCMICR) were originally coded as binary variables (12, 23).

To quantify tau pathology, the Braak stage (NACCBRAA) was recoded into an ordered categorical variable. Braak stages were recategorized into four ordinal groups based on standard staging conventions: stages 0 (Braak = 0) were labeled as “B0” (no neurofibrillary pathology); stages 1–2 as “B1” (trans-entorhinal); stages 3–4 as “B2” (limbic); and stages 5–6 as “B3” (isocortical involvement). We used the neuritic plaque density variable (NACCNEUR), which was recorded in severity levels ranging from 0 to 3, to account for neuritic amyloid plaque pathology. We isolated participants with moderate-to-severe tau burden (Braak stage B2 or B3) and none/ low neuritic plaque density (NACCNEUR score of 0 or 1) for our analytical sample. This combination reflects individuals with predominately tau-positive neurofibrillary tangle pathology in the context of none/ low neuritic plaques, which may represent an alternative neurodegenerative pathway that does not follow the traditional amyloid cascade hypothesis.

### Neuropathology exclusions in intermediate/high neurofibrillary tau (T+) and low or no neuritic plaque (T+A−) cohort

To isolate a neuropathologically homogenous sample and reduce confounding from comorbid pathological processes, we applied sequential exclusion criteria to remove participants with non-AD pathologies aligned with previous studies (12, 24). We first excluded individuals with vascular pathology (e.g., cerebral autosomal dominant arteriopathy with subcortical infarcts and leukoencephalopathy (CADASIL)) based on NPPVASC and NPPATH10 variables. We next excluded participants with frontotemporal lobar degeneration (FTLD), prion disease, tauopathies, and related proteinopathies by removing those with positive codes (value = 1) for a comprehensive set of neuropathological flags, including NPPFTLD, NPCFTLD, NPPPRION, NPCPRION, NPFTDTAU, NPFTDTDP, NPFTDNO, NPFTDSPC, NPFTDT2 through NPFTDT10, and NPOFTD through NPOFTD5. Similarly, cases positive for Pick’s disease (NACCPICK), CBD (NACCCBD), progressive supranuclear palsy (NACCPROG), and hippocampal sclerosis (NPPHIPP, NPCHIPP) were removed. We further excluded participants with amyotrophic lateral sclerosis motor neuron disease (NPALSMND), chromosomal abnormalities (NPCHROM, NACCDOWN), and a wide array of other rare or undefined neuropathologies (e.g., NPPDXA–NPPDXN, NPPOTH1–NPCOTH3, and NPTDPA–NPTDPE). This process yielded a final analytic cohort of 579 participants, reflecting a conservative sample with reduced neuropathological heterogeneity, aligned with our hypothesis.

### Harmonized cognitive domain score measurements

The harmonized cognitive domain scores based on neuropsychological assessment developed for NACC and other clinical and population based cohorts have been previously described (25). In brief, a multidisciplinary panel reviewed cognitive test items and classified them into core cognitive domains, including memory, executive function, language, and visuospatial ability, while some items were not assigned to any domain. These classifications informed the use of confirmatory factor analysis conducted on a pooled dataset to establish latent cognitive constructs. Item response theory methods were applied to derive factor scores and standard errors using anchor items shared across studies. A visuospatial harmonized cognitive score was not derived for NACC since there was limited item data related to that document available at the NACC visits. The harmonized cognitive scores outcomes are z-scored ranges.

### NACC harmonized cardiovascular disease risk score

To standardize cardiovascular risk factor data across multiple cohorts, including NACC, a harmonized workflow was developed and is described elsewhere (26). The harmonization workflow utilized participants’ self-reported history of heart disease, hypertension (HTN), and diabetes mellitus (DM). In addition, body mass index (BMI) from the most recent study visit was included. Reports of any cardiovascular condition, regardless of severity or timing, were considered sufficient for inclusion. The four indicators, heart disease, HTN, DM, and BMI, were integrated into a composite cardiovascular risk score using principal component analysis. The first principal component, which captured the greatest variance among the four indicators, was retained as the primary risk score. Full documentation of the harmonization process is available in the Cardiovascular Harmonization Workflow README file (27), with methodology further described by Lee et al. (27) and the Alzheimer’s Disease Sequencing Project Phenotype Harmonization Consortium (ADSP-PHC) (26).

## Statistical Analysis

### Logistic Regression

To investigate the relationship between vascular neuropathologies and clinical dementia status/cognitive impairment, we fit separate multivariable logistic regression models for each vascular neuropathology. The binary outcome variable, “any dementia,” was derived by recoding the Clinical Dementia Rating Global Score (CDR) where 0 indicated no dementia/ cognitive impairment and > 0 indicating the presence of cognitive impairment/dementia. Each of the five models examined a different vascular marker—arteriolosclerosis, atherosclerosis, CAA, gross infarcts, and microinfarcts—as the primary predictor. All models were adjusted for age at death, sex, education, and *APOE* ε4 status. Results are reported as odds ratios (OR) with corresponding 95% confidence intervals (CI) and p-values.

### Ordinal Logistic Regression

To evaluate associations between vascular neuropathology and CDR stages, we used ordinal logistic regression models with global CDR as the outcome. For modeling purposes, participants with global CDR scores of 0 or 0.5 were grouped together and coded as 0, representing the lowest level of impairment, while higher scores reflected increasing dementia severity. As with the logistic models, five separate ordinal logistic regressions were run, one per vascular pathology. All the models were adjusted for age at death, sex, education, and *APOE* ε4 carrier status. Results are reported as OR with 95% CI and p-values.

### Multivariable Linear Regression

We conducted domain-specific multivariable linear regression models to examine the cross-sectional associations between vascular neuropathologies and cognitive scores proximate to death. Outcomes included memory, executive function, language, and CDR-Sum of Boxes (CDR-SOB). For each outcome, five separate models were estimated—one per vascular predictor—adjusting for age at death, sex, years of education, and *APOE* ε4 status. Results are reported as standardized beta coefficients (β) with 95% CI.

### Sensitivity Analysis: Adjustment for Cardiovascular Risk

To address potential confounding by systemic cardiovascular health, we repeated the above analyses with additional adjustment for a harmonized cardiovascular risk score derived from the ADSP-PHC (26). Each outcome was modeled separately with the same five vascular predictors and covariates (age, sex, education, *APOE* ε4, and cardiovascular risk).

### Sensitivity Analysis: Neuritic Amyloid-Negative Subgroup

To isolate the impact of vascular neuropathology in the absence of neurtic plaques, we restricted analyses to participants with elevated neurofibrillary tau pathology (Braak stages B2 or B3) and no neuritic plaques (NACCNEUR = 0). Models were otherwise identical in structure and covariate adjustment.

### Sensitivity Analysis: Adjusting for Tau and Neuritic Amyloid Plaque Pathologies

To evaluate the independence of vascular effects from Alzheimer’s hallmark pathologies, we adjusted for both Braak stage (B2 vs. B3) and neuritic plaque burden (C0 vs. C1). Linear regression models were again stratified by cognitive outcome and included all prior described covariates.

### Linear Mixed-Effects Models

Longitudinal associations between neuropathologies and cognitive decline were examined using linear mixed-effects models, with separate models per outcome. Time was modeled continuously as years to death with 0 being death. Each model included an interaction between neuropathology and time to test whether cognitive decline varied by pathology. Models adjusted for sex, education, *APOE* ε4 status, and included random intercepts and slopes for years to death at the participant level. Interaction β estimates reflect the difference in rate of cognitive decline prior to death by pathology status and are reported with 95% CI. Model example: (cognitive domain ~ years prior to death * vascular neuropathology + sex + education + APOE e4 + (years prior to death | participant identifier).

All statistical analyses were conducted using R version 4.2.3. All p-values are two-sided, and the statistical significance was set at p-value < 0.05 for all demographic comparisons.

### Data availability

All data used in the current analyses is available for download from the NACC (https://www.alz.washington.edu/).

## Results

Among the 579 decedents with none/low neuritic plaques and intermediate/high neurofibrillary tau pathology, the majority were White (95.7%), with a mean age at death of 88.4 years and nearly half identified as male (49.4%) (Table 1). The majority of the sample had a Consortium to Establish a Registry for Alzheimer’s Disease score of 1 (59.9%) and were classified at Braak stage B2 (82.4%). Vascular neuropathologies were prevalent with atherosclerosis (43.2%) and arteriolosclerosis (40.2%) being the most common. Additionally, 29.5% were *APOE* ε4 carriers. In terms of cognitive status, 30.9% had no dementia/ cognitive impairment (CDR = 0) and the mean CDR-SOB was 5.34.

Clinical diagnoses are provided in Supplemental Table 1. The primary etiologic diagnosis in this analytical sample was AD (39%), despite lacking intermediate and advanced neuritic plaques pathology. An additional 32% were classified as cognitively normal at their visit most approximate to death, suggesting substantial clinical heterogeneity within this subgroup. Vascular dementia or vascular brain injury accounted for 6% of diagnoses, while neurodegenerative conditions such as LBD (13.5%) and various non-AD pathologies were also represented in small percentages.

Arteriolosclerosis (OR = 1.66, 95% CI: 1.12–2.49, *p* = 0.01), atherosclerosis (OR = 1.57, 95% CI: 1.05–2.36, *p* = 0.03), gross infarcts (OR = 4.05, 95% CI: 2.34–7.41, *p* < 0.001), and microinfarcts (OR = 1.97, 95% CI: 1.27–3.09, *p* = 0.003) were all significantly associated with any dementia/ cognitive impairments based on a global CDR greater than 0 (Table 2). CAA did not show a significant association (OR = 1.12, 95% CI: 0.67–1.92, *p* = 0.66). Unlike the logistic regression results, arteriolosclerosis (*p* = 0.77), atherosclerosis (*p* = 0.65), CAA (0.48), and microinfarcts (*p* = 0.07) were not associated with a greater odds across dementia stages using the ordinal values from the global CDR (Table 3). However, gross infarcts remained significantly associated with dementia severity (OR = 1.99, 95% CI: 1.30–3.05, *p* < 0.01).

At the visit closest to death, microinfarcts were significantly associated with poorer memory (*β* =−0.28, 95% CI: −0.51 – −0.05, *p* = 0.02), executive function (*β* =−0.24, 95% CI: −0.44 – −0.04, *p* = 0.02), and language performance (*β* =−0.21, 95% CI: −0.38 – −0.04, *p* = 0.02, Table 4). Gross infarcts were also linked to worse language scores (*β* =−0.20, 95% CI: −0.40 – −0.01, *p* = 0.04) and showed a strong association with worse CDR-SOB scores (*β* = 1.87, 95% CI: 0.72–3.02, *p* < 0.01, Table 4).

When controlling for harmonized cardiovascular disease risk, arteriolosclerosis was significantly associated with worse language performance (*B*=−0.17, 95% CI: −0.32 – −0.01, *p* = 0.03, Supplementary Table 2). Microinfarcts remained significantly associated with poorer memory (*β* =−0.25, 95% CI: −0.49 to −0.01, *p* = 0.04), executive function (*β* =−0.25, 95% CI: −0.45 – −0.04, *p* = 0.02), and language scores (*β* =−0.21, 95% CI: −0.39 – −0.04, *p* = 0.02, Supplementary Table 2). When removing participants with low neuritic plaques, the associations between microinfarcts and cognition increased and remained significant: memory (*β* =−0.41, 95% CI: −0.92 – −0.11, *p* = 0.01), executive function (*β* =−0.48, 95% CI: −0.77 – −0.19, *p* < 0.01), language (*β* =−0.27, 95% CI: −0.51 – −0.02, *p* = 0.03), and an increase in CDR sum of boxes (*β* = 1.46, 95% CI: 0.13–2.80, *p* = 0.03, Supplementary Table 3). In fully adjusted linear regression models that controlled for both neuritic plaque burden stage and Braak stage, microinfarcts remained significantly associated with poorer performance across all three cognitive domains and greater dementia severity (Supplemental Table 4).

**Figure 1:**
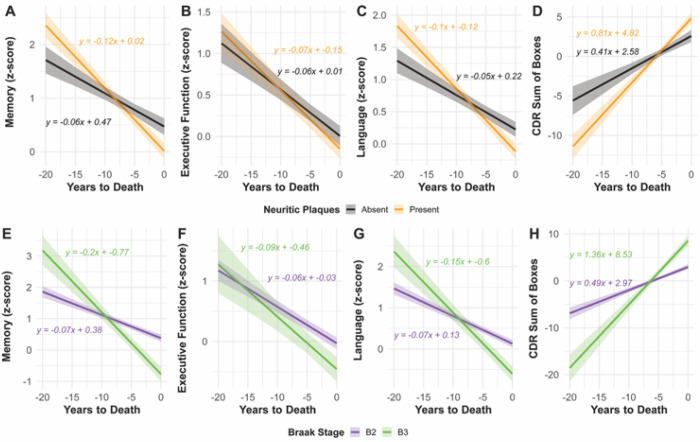
Trajectories of cognitive decline by neuritic plaque status and Braak stage among neurofibrillary tangle predominant decedents Longitudinal cognitive performance trajectories by years to death for individuals with intermediate-to-high neurofibrillary burden and low/absent neuritic plaque pathology. Panels A–D depict the interaction between neuritic plaque presence (NACCNEUR) and time to death across four cognitive outcomes: Memory (A), Executive Function (B), Language (C), and Global Cognition (Clinical Dementia Rating Sum of Boxes (CDR-SOB); D). Neuritic plaque presence is associated with faster decline across all domains. Panels E–H display the interaction between Braak stage (B2 vs. B3) and time to death across the same outcomes: Memory (E), Executive Function (F), Language (G), and Global Cognition (H). Steeper slopes were observed among individuals with Braak stage B3, particularly for memory and global cognition. Regression line equations are annotated for each group. Shaded regions represent 95% confidence intervals. All models are adjusted for age at death, sex, education, and apolipoprotein (*APOE)* ε4 status and account for random intercepts and random slopes for each participant.

Linear mixed-effects models revealed significant associations between neuritic plaques and neurofibrillary tau with the rate of cognitive decline in the years preceding death (Fig. 1; Supplemental Table 5). In model 1, the interaction between time to death and low neuritic plaque burden (C1) compared to no neuritic plaque burden (C0) was significantly associated with faster decline in memory (β = −0.05, 95% CI: −0.08 – −0.03, p < 0.01), language (β = −0.04, 95% CI: −0.06 – −0.03, p < 0.01), and increased CDR-SOB scores (β = 0.40, 95% CI: 0.25–0.56, p < 0.01), but not with executive function (p = 0.13) (Fig. 1A-D). In model 2, Braak stage (B3) compared to B2 exhibited steeper longitudinal declines in memory (β = −0.12, 95% CI: −0.16 – −0.09, p < 0.01) and language (β = −0.08, 95% CI: −0.11 – −0.06, p < 0.01), and with greater increase in global impairment (CDR-SOB; β = 0.86, 95% CI: 0.66–1.06, p < 0.01) (Fig. 1E-G). Model 3 included both neuritic plaques variable and Braak stage variable simultaneously, and results were similar to individual models 1 and 2 (Supplemental Table 5).

We examined whether the association between time to death and cognitive trajectories differed based on the presence of specific vascular neuropathologies (Table 5). Atherosclerosis was significantly associated with slower declines in executive function (β = 0.02, 95% CI: 0.00–0.04, *p* = 0.02) and increases in global impairment over time (CDR Sum of Boxes: β = −0.21, 95% CI: −0.36 – −0.05, *p* = 0.01). CAA also showed a significant interaction with time, associated with worsening CDR-SOB (β = 0.40, 95% CI: 0.20–0.59, *p* < 0.01), although no associations were observed with domain-specific outcomes. Gross infarcts were significantly associated with faster language decline over time (β = −0.02, 95% CI: −0.05 – −0.00, *p* = 0.02).

## Discussion

The objective of this study was to investigate whether vascular neuropathologies contribute to cognitive impairment among decedents with intermediate-to-high tau pathology and sparse neuritic plaque pathology. This biomarker profile has been understudied, with only one other study investigating the role of vascular burden on A-T + or A-N + patients (28). In this study using autopsy-confirmed data from the NACC cohort, we examined the independent associations between arteriolosclerosis, atherosclerosis, CAA, infarcts, and microinfarcts with global and domain-specific cognitive outcomes and found robust associations between microvascular pathology and cognitive decline in this unique group of sparse amyloid pathology. These findings highlight a possible distinct vascular-neurodegenerative pathway that operates independently of classical AD mechanisms.

The increasing evidence of a tangle-predominant or tangle-only dementia has led to the acceptance of PART as a distinct entity apart from AD (9). While our analyzed cohort is defined by no-to-sparse neuritic plaques and does not account for diffuse amyloid plaques due to coding and sample size constraints, our cohort best resembles those used in studies focused on PART (9). Indeed, 30.9% of our cohort did not display cognitive impairment clinically (as defined by global CDR) which is in line with past autopsy studies reporting no significant antemortem cognitive impairment in individuals with PART or Aβ-independent tauopathy (29–31). Our study particularly complements Besser et al. 2017 findings that increasing Braak stage and history of stroke was associated with cognitive impairment in individuals with neurofibrillary tangles and sparse neuritic plaques. Our study goes further by highlighting the robust association of microinfarctions to poor performance in specific cognitive domains (memory, executive function, and language) cross-sectionally most proximal to death but not longitudinally.

The deleterious effects of comorbidities such as neurovascular pathologies including gross infarcts, and microinfarctions, on cognition independent of and in association with other neurodegenerative pathologies are well established (12, 32–34). Of note, an autopsy study of individuals with AD and individuals with Aβ-independent tauopathy (i.e., PART) report that while the incidence of cerebrovascular disease and hemorrhages are greater in their AD group, both groups have similar frequency of gross infarcts (microinfarcts were not evaluated) (31). Both groups in their study also experienced significant cognitive impairment in the presence of cerebrovascular disease. Similarly, in our analysis, gross infarcts were linked to higher dementia severity and worse language function. We further found that microinfarcts were consistently associated with poorer performance in memory, executive function, and language, even after adjusting for demographic factors, *APOE* ε4 status, and cardiovascular risk. Notably, these associations persisted—and in some cases are strengthened—in our sensitivity analyses which accounted for the effects of cardiovascular disease, neurofibrillary tau burden, and even in the absence of neuritic plaque burden. Our study overall supports that vascular neuropathology, particularly microinfarcts, play a central role in cognitive decline in the context of Aβ-independent tauopathy.

### Clinical Significance and Importance

These findings have important clinical implications for how we conceptualize, diagnose, and manage cognitive impairment in aging populations. First, they underscore the need to look beyond amyloid when evaluating causes of cognitive decline, especially in individuals who may fall under the SNAP or PART categories. Recent in vivo evidence has suggested that co-pathology, in particular vascular pathologies, may exert a strong negative impact on cognition in participants who have less neurofibrillary tau pathology (35); however, these findings are in consideration of the updated biological definition of AD compared to the clinical (7). Plasma and neuroimaging biomarkers that capture microvascular injury and tau burden—but not necessarily amyloid—may be critical for risk stratification in these individuals. Second, our results support growing calls for the development of vascular-targeted interventions, including antihypertensive, antithrombotic, or anti-inflammatory strategies, as potential avenues to prevent or slow cognitive decline in older adults, regardless of amyloid status. For example, individuals with two or more microinfarcts—regardless of antihypertensive use—were more likely to develop dementia in the absence of AD pathology (36). Finally, this work reinforces the need for greater inclusion of biologically heterogeneous participants in clinical trials and observational studies. Individuals who are amyloid-negative but tau-positive may be at substantial risk for cognitive decline driven by non-AD pathways and excluding them may overlook opportunities for tailored treatment. Integrating vascular health assessments into diagnostic frameworks and trial eligibility criteria could better capture the full spectrum of late-life cognitive impairment and improve real-world generalizability of therapeutic approaches.

### Limitations and Strengths

Several limitations should be acknowledged. First, the cross-sectional nature of the primary analyses limits our ability to infer temporality or causality between vascular neuropathology and cognitive outcomes. While our findings are biologically plausible and supported by neuropathological literature, longitudinal confirmation at the temporal level to track time of onset of microinfarcts specifically is needed. Second, despite the harmonized protocols of the NACC dataset, differences in data collection across sites and incomplete biomarker availability (e.g., for plasma amyloid/tau or longitudinal cognitive trajectories) may introduce measurement variability. Third, although we adjusted for key confounders such as age, sex, education, *APOE* ε4 status, and cardiovascular risk, residual confounding from unmeasured lifestyle, genetic, or environmental factors remains possible. Finally, the sample was predominantly non-Hispanic White, which limits generalizability to other racial ethnicities disproportionately affected by vascular disease and cognitive decline (37).

Despite these limitations, our study has several important strengths. We leveraged a large, well-characterized autopsy dataset with harmonized neuropathological and cognitive data, allowing for precise stratification of neuritic plaque-negative, neurofibrillary tau-positive individuals—a biologically distinct and underrepresented group in AD research. The inclusion of multiple vascular markers, both focal (e.g., microinfarcts) and systemic (e.g., arteriolosclerosis), enabled a comprehensive assessment of vascular burden. Additionally, we incorporated multiple cognitive domains and adjusted for cardiovascular disease risk in sensitivity analyses, enhancing the robustness of our findings. Our approach reflects real-world complexity in aging brains and contributes valuable insight into non-amyloid drivers of cognitive decline.

### Future Directions

Future research should explore these vascular-cognitive relationships using longitudinal designs to better characterize the temporal dynamics between vascular pathology and cognitive decline in amyloid-negative individuals. In vivo biomarkers of microvascular injury, such as diffusion magnetic resonance imaging or plasma markers of endothelial dysfunction, may help identify at-risk individuals prior to death and facilitate early intervention. Moreover, expanding this work to more racially and ethnically representative populations is essential to understanding how social determinants of health including lifestyle risks may interact with vascular and tau pathology. Finally, incorporating multimodal data—including genomics, metabolomics, and inflammation profiles—could provide deeper mechanistic insight and identify novel therapeutic targets for individuals on non-AD pathways to dementia.

## Supplementary Material

1

## Figures and Tables

**Table 1:** Demographics, neuropathology, and clinical characteristics of neurofibrillary tangle predominant decedents

	Overall (N = 579)
Age at Death, years Mean (SD)	88.4 (8.7)
Male, n (%)	286 (49.4)
Education, years Mean (SD)	16.3 (8.3)
Missing, n (%)	4 (0.0)
Hispanic Ethnicity, n (%)	3 (0.01)
Missing, n (%)	576 (99.4)
Race, n (%)	
White	554 (95.7)
Black or African American	18 (3.1)
American Indian or Alaska Native	1 (0.2)
Native Hawaiian or Other Pacific Islander	0 (0)
Asian	2 (0.3)
Unknown	2 (0.3)
Neuritic Plaques	
CERAD 0, n (%)	232 (40.1)
CERAD 1, n (%)	347 (59.9)
Braak Stage	
B2, n (%)	477 (82.4)
B3, n (%)	102 (17.6)
Arteriolosclerosis, n (%)	233 (40.2)
Atherosclerosis, n (%)	250 (43.2)
CAA, n (%)	104 (18.0)
Missing, n (%)	6 (1)
Microinfarcts, n (%)	163 (28.2)
Gross Infarcts/ Lacunes, n (%)	115 (19.9)
*APOE* ε4 carrier status, n (%)	171 (29.5)
Missing, n (%)	49 (8.5)
Memory Mean (SD)	−0.1 (1.1)
Missing, n (%)	176 (30.4)
Executive Function Mean (SD)	−0.1 (0.8)
Missing, n (%)	265 (45.8)
Language Mean (SD)	−0.1 (0.8)
Missing, n (%)	200 (34.5)
CDR Global, n (%)	
0	179 (30.9)
0.5	156 (26.9)
1	84 (14.5)
2	77 (13.3)
3	83 (14.3)
CDR Sum of Boxes Mean (SD)	5.34 (6.1)

This table presents the demographic, neuropathological, and cognitive characteristics of 579 decedents with none/low neuritic amyloid plaques and intermediate/high tau pathology. Variables include age at death, sex, years of education, ethnicity, and race. Neuropathological measures include CERAD neuritic amyloid plaque scores, Braak staging for tau pathology, arteriolosclerosis, atherosclerosis, cerebral amyloid angiopathy (CAA), microinfarcts, gross infarcts/lacunes, and *APOE* (apolipoprotein) ε4 carrier status. Cognitive assessments include domain-specific scores for memory, executive function, and language, as well as global cognitive status using the Clinical Dementia Rating (CDR) scale and the CDR Sum of Boxes. Data are reported as means with standard deviations (SD) for continuous variables and counts with percentages for categorical variables. Missing data are noted where applicable.

**Table 2:** Association between vascular neuropathologies and any cognitive impairment stage (global CDR > 0)

Predictor	OR (95% CI))	p
Arteriolosclerosis	**1.66 (1.12–2.49)**	**0.01**
Atherosclerosis	**1.57 (1.05–2.36)**	**0.03**
CAA	1.12 (0.67–1.92)	0.66
Gross Infarcts	**4.05 (2.34–7.41)**	**< 0.001**
Microinfarcts	**1.97 (1.27–3.09)**	**0.003**

This table displays the odds ratios (OR) with 95% confidence intervals (CI) and corresponding p-values for the association between vascular neuropathologies and the presence of any dementia, defined as a global Clinical Dementia Rating (CDR) greater than 0. Vascular neuropathologies examined include arteriolosclerosis, atherosclerosis, cerebral amyloid angiopathy (CAA), gross infarcts, and microinfarcts. Statistical significance was determined using logistic regression models, with p-values < 0.05 considered significant.

**Table 3:** Cross-validation testing associations between vascular neuropathologies and dementia stages using global CDR

Predictor	OR (95% CI))	p
Arteriolosclerosis	1.06 (0.73–1.54)	0.77
Atherosclerosis	1.09 (0.75–1.59)	0.65
CAA	1.17 (0.75–1.83)	0.48
Gross Infarcts	**1.99 (1.30–3.05)**	**< 0.01**
Microinfarcts	1.43 (0.97–2.13)	0.07

This table presents the results of an ordinal logistic regression analysis assessing the associations between vascular neuropathologies and dementia severity stages, as measured by the global Clinical Dementia Rating (CDR). Odds ratios (OR) with 95% confidence intervals (CI) and corresponding p-values are reported for arteriolosclerosis, atherosclerosis, cerebral amyloid angiopathy (CAA), gross infarcts, and microinfarcts. The model evaluates the likelihood of progressing to higher dementia stages based on the presence of each vascular pathology. Cross-validation was used to test the stability and generalizability of the associations. Statistical significance was defined as p < 0.05

**Table 4:** Association between vascular neuropathologies and cross-sectional cognitive domain and dementia scores proximal to death

	Memory		Executive Function		Language		CDR Sum of Boxes	
Predictor	β (95% CI)	p	β (95% CI)	p	β (95% CI)	p	β (95% CI)	p
Arteriolosclerosis	−0.12 (−1.33–0.09)	0.26	−0.11 (−0.29–0.07)	0.24	−0.14 (−0.29–0.01)	0.06	0.08 (−0.86–1.01)	0.87
Atherosclerosis	−0.10 (−0.32–0.12)	0.35	−0.02 (−0.21–0.16)	0.81	0.08 (−0.08–0.24)	0.33	0.07 (−0.92–1.05)	0.89
CAA	−0.17 (−0.45–0.11)	0.23	−0.06 (−0.29–0.18)	0.64	0.01 (−0.20–0.22)	0.93	0.87 (−0.35 – 2.09)	0.16
Gross Infarcts	−0.24 (−0.51–0.02)	0.07	−0.10 (−0.33–0.13)	0.39	**−0.20 (−0.40 – −0.01)**	**0.04**	**1.87 (0.72–3.02)**	**< 0.01**
Microinfarcts	**−0.28 (−0.51 – −0.05)**	**0.02**	**−0.24 (−0.44 – −0.04)**	**0.02**	**−0.21 (−0.38 – −0.04)**	**0.02**	0.93 (−0.11–1.97)	0.08

This table presents the results of linear regression analyses examining the associations between vascular neuropathologies and cognitive domain scores (memory, executive function, and language), as well as global dementia severity measured by the Clinical Dementia Rating (CDR) Sum of Boxes, in the period proximal to death. The table reports beta coefficients (β) with 95% confidence intervals (CI) and corresponding p-values for each vascular pathology, including arteriolosclerosis, atherosclerosis, cerebral amyloid angiopathy (CAA), gross infarcts, and microinfarcts. Negative β values indicate worse cognitive performance associated with the presence of the pathology. For the CDR Sum of Boxes, higher β values reflect greater dementia severity. Statistical significance was defined as p < 0.05.

**Table 5:** Interaction effects between vascular neuropathologies and years to death on longitudinal cognitive outcomes

	Memory		Executive Function		Language		CDR Sum of Boxes	
Predictors	β (95% CI)	p	β (95% CI)	p	β (95% CI)	p	β (95% CI)	p
years to death × arteriolosclerosis	0.01 (−0.01–0.03)	0.29	0.00 (−0.02–0.02)	0.99	0.01 (−0.01–0.02)	0.32	−0.08 (−0.22–0.06)	0.24
years to death × atherosclerosis	0.00 (−0.02–0.02)	0.98	**0.02 (0.00–0.04)**	**0.02**	0.00 (−0.01–0.02)	0.69	**−0.21 (−0.36 – −0.05)**	**0.01**
years to death × CAA	−0.02 (−0.05–0.01)	0.28	−0.01 (−0.04–0.01)	0.32	−0.01 (−0.03–0.01)	0.48	**0.40 (0.20–0.59)**	**< 0.01**
years to death × gross infarcts	−0.01 (−0.04–0.02)	0.45	0.01 (−0.02–0.03)	0.62	**−0.02 (−0.05 – −0.00)**	**0.02**	0.14 (−0.06–0.34)	0.16
years to death × microinfarcts	0.00 (−0.02–0.03)	0.77	0.00 (−0.02–0.02)	0.81	−0.00 (−0.02–0.02)	0.86	−0.10 (−0.27–0.07)	0.24

Linear mixed-effects models were used to evaluate the interaction between time to death and five vascular neuropathologies—arteriolosclerosis, atherosclerosis, cerebral amyloid angiopathy (CAA), gross infarcts, and microinfarcts—on cognitive trajectories. Outcomes included memory, executive function, language, and global cognition (CDR Sum of Boxes). Interaction terms (years to death × vascular neuropathology) reflect differences in the rate of cognitive decline associated with each neuropathological marker. Estimates reflect the slope difference in cognitive change over time associated with each pathology group. Beta coefficients (β), 95% confidence intervals (CI), and p-values are reported for each domain. Significant associations (p < 0.05) are bolded.

## Data Availability

The HABS-HD data is available upon request at: https://apps.unthsc.edu/itr/

## Abbreviations

A+: amyloid-β (Aβ) plaques
T+: neurofibrillary tau tangles
AD: Alzheimer’s disease
A: amyloid
T: tau
N: neurodegeneration
CAA: cerebral amyloid angiopathy
NACC: National Alzheimer’s Coordinating Center
Aβ: amyloid-β
LBD: Lewy body disease
ADNC: Alzheimer’s disease neuropathologic change
PART: primary age-related tauopathy PART)
SNAP: suspected non-Alzheimer’s pathophysiology
ADRC: Alzheimer’s Disease Research Centers
MCI: mild cognitive impairment
APOE: apolipoprotein
CAA: cerebral amyloid angiopathy
CADASIL: cerebral autosomal dominant arteriopathy with subcortical infarcts and leukoencephalopathy
FTLD: frontotemporal lobar degeneration
HTN: hypertension
DBT: diabetes
BMI: body mass index
ADSP-PHC: Alzheimer’s Disease Sequencing Project Phenotype Harmonization Consortium
CDR: clinical dementia rating
OR: odds ratio
CI: confidence intervals
CDR-SOB: CDR-Sum of Boxes
β: beta coefficients
