# Supplementary material for: Microinfarcts are Associated with Cognitive Impairment in Neurofibrillary Tangle Predominant Decedents: Evidence from the NACC Autopsy Cohort
Source: Res Sq. 2025 Jul 14:rs.3.rs-7036276. Preprint. [Version 1] doi: 10.21203/rs.3.rs-7036276/v1 (PMC12288546; doi:10.21203/rs.3.rs-7036276/v1)
Supplement: 1 [file NIHPPrs7036276v1-supplement-1.pdf]

Supplementary Files

- SupplementalTables.docx
